# Impact of platelet lysate on immunoregulatory characteristics of equine mesenchymal stromal cells

**DOI:** 10.3389/fvets.2024.1385395

**Published:** 2024-04-24

**Authors:** Julia Moellerberndt, Sabine Niebert, Kerstin Fey, Alina Hagen, Janina Burk

**Affiliations:** ^1^Equine Clinic (Surgery, Orthopedics), Justus-Liebig-University Giessen, Giessen, Germany; ^2^Institute of Physiology, Pathophysiology, and Biophysics, University of Veterinary Medicine Vienna, Vienna, Austria; ^3^Equine Clinic (Internal Medicine), Justus-Liebig-University Giessen, Giessen, Germany

**Keywords:** platelet lysate, MSc, equine, IFN-γ, TNF-α, IL-1β, IL-10, culture medium

## Abstract

Multipotent mesenchymal stromal cells (MSC) play an increasing role in the treatment of immune-mediated diseases and inflammatory processes. They regulate immune cells via cell-cell contacts and by secreting various anti-inflammatory molecules but are in turn influenced by many factors such as cytokines. For MSC culture, platelet lysate (PL), which contains a variety of cytokines, is a promising alternative to fetal bovine serum (FBS). We aimed to analyze if PL with its cytokines improves MSC immunoregulatory characteristics, with the perspective that PL could be useful for priming the MSC prior to therapeutic application. MSC, activated peripheral blood mononuclear cells (PBMC) and indirect co-cultures of both were cultivated in media supplemented with either PL, FBS, FBS+INF-γ or FBS+IL-10. After incubation, cytokine concentrations were measured in supernatants and control media. MSC were analyzed regarding their expression of immunoregulatory genes and PBMC regarding their proliferation and percentage of FoxP3+ cells. Cytokines, particularly IFN-γ and IL-10, remained at high levels in PL control medium without cells but decreased in cytokine-supplemented control FBS media without cells during incubation. PBMC released IFN-γ and IL-10 in various culture conditions. MSC alone only released IFN-γ and overall, cytokine levels in media were lowest when MSC were cultured alone. Stimulation of MSC either by PBMC or by PL resulted in an altered expression of immunoregulatory genes. In co-culture with PBMC, the MSC gene expression of COX2, TNFAIP6, IDO1, CXCR4 and MHC2 was upregulated and VCAM1 was downregulated. In the presence of PL, COX2, TNFAIP6, VCAM1, CXCR4 and HIF1A were upregulated. Functionally, while no consistent changes were found regarding the percentage of FoxP3+ cells, MSC decreased PBMC proliferation in all media, with the strongest effect in FBS media supplemented with IL-10 or IFN-γ. This study provides further evidence that PL supports MSC functionality, including their immunoregulatory mechanisms. The results justify to investigate functional effects of MSC cultured in PL-supplemented medium on different types of immune cells in more detail.

## Introduction

Platelet lysate (PL) is a promising alternative to fetal bovine serum (FBS) as cell culture supplement for mesenchymal stromal cells (MSC). In previous studies, we were able to demonstrate that equine PL, produced by using the buffy coat method, promotes equine MSC proliferation, as well as their genetic stability and pro-angiogenic potential (1, 2). MSC characteristics such as differentiation capacity and expression of surface markers were similar after cultivating the cells with PL or FBS (1). PL contains a diversity of nutrients and growth factors such as TGF-β and PDGF (1, 3). However, even while using a leukocyte-poor platelet concentrate for PL production (1), the resulting PL also contains a variety of cytokines including IFN-γ, IL-10, IL-1β, IL-4, IL-6 and TNF-α (4). Cytokines are a group of peptides that regulate the proliferation and differentiation of cells. They play an important role as inflammatory mediators, growth factors and in immune reactions. They are secreted by various populations of cells, interacting among themselves and with other cells. Thus, it is to be expected that the cytokines in PL also influence MSC.

MSC are a mixture of cells of mesenchymal origin (excluding hematopoietic or endothelial cells) (5), which can differentiate into various cell populations such as cartilage, fat or bone cells and should be characterized by a specific immunophenotype (6). In case of tissue destruction, MSC are released into the blood circulation and migrate to the lesion, steered by chemokines, and priming the microenvironment for regeneration by secreting various molecules (7, 8).

For therapeutic use, MSC can be isolated from various body tissues such as fat or bone marrow (9, 10). In equine musculoskeletal diseases such as osteoarthritis (11) and tendinopathies (12) allogeneic MSC products have been authorized as therapeutics in the EU. In human medicine, MSC therapies were established for immune-related diseases such as graft-vs.-host disease (13, 14), among many other possible applications. The research focus regarding MSC modes of action has shifted from differentiation to immunomodulatory activities. Earlier studies showed that the use of MSC in mammals with inflammatory diseases significantly reduces T cell proliferation (15–18). Important immunomodulatory molecules secreted by MSC are nitric oxide (NO), prostaglandin E2 (PGE2), transforming growth factor-β (TGF-β), galactin 9 and TNF-stimulated gene 6 protein (TSG6) (19–21). PGE2 secretion leads to an increased expression of IDO, NOS, IL-6 and COX2, which serve as supporting paracrine signals for the suppression of T cell proliferation by MSC (22, 23). In addition, MSC can support the polarization of macrophages from a pro-inflammatory to an anti-inflammatory phenotype by producing immunosuppressive molecules such as PGE2 (24) or TSG6 (25).

MSC priming with pro-inflammatory cytokines prior to their therapeutic use could increase their efficiency *in vitro* (18). Cytokines such as interferon-γ (IFN-γ) induce the expression of IDO and secretion of PGE2 (16, 26). As described above, naïve MSC can already inhibit T cell proliferation, but not T cell effector function (27). After inflammatory priming of the MSC, PGE2 secretion is increased and IDO expressed, which then inhibits T cell effector function (28). In addition, *in vitro* priming with TNF-α increased MSC migration by upregulating various chemokine receptors (29), which could be beneficial because only low percentages of MSC reach the site of action after systemic application (30). The positive effects of priming MSC with cytokines depend on concentration and incubation time. For example, high doses of IFN-γ (25 ng/ml or 100 ng/ml) can promote T cell activation through induction of MHCI and -II antigens (31, 32), whereas lower doses (5 ng/ml of TNFα and IFNγ) promoted the expression of immunoregulatory genes and MHCII and CD40 but not co-stimulatory molecules (33).

Our hypothesis was that PL would promote MSC immunoregulatory activity in a similar manner as FBS with cytokine supplementation. For this purpose, we assessed MSC and peripheral blood mononuclear cells (PBMC) cultured alone and in indirect co-culture in media supplemented with PL or standard FBS, as well as FBS supplemented with the most abundant cytokines in the PL, IFN-γ and IL-10. The aim of the study was to investigate if culturing MSC with PL has an impact on their immunoregulatory mechanisms, due to the cytokines contained. If so, PL could help to prime MSC based on its naturally occurring mix of ingredients, potentially yielding good immunomodulatory effects but low immunogenicity of the MSC.

## Materials and methods

### Cell isolation and PL production

PBMC were isolated from one horse. Whole blood was collected at several times over a period of one year, as approved by the responsible authority (regional council Giessen, A17/2018). Ten ml whole blood freshly collected in lithium-heparin tubes (S-Monovette Lithium Heparin LH, Sarstedt, Nümbrecht, Germany) were diluted with an equal amount of PBS. The diluted blood was then layered onto 15 ml of Ficoll Paque Premium (Cytiva, Marlborough, Massachusetts, USA) and centrifuged at 400 g at RT for 40 min, without brakes. The layer of white blood cells was harvested and mixed with 20 ml of PBS, then centrifuged again at 400 g at RT for 5 min. The supernatant was aspirated and the washing step was repeated. The peripheral blood mononuclear cells (PBMC) were then counted and frozen as aliquots in cryomedium consisting of Dulbecco's modified Eagle's medium (DMEM, 1 g/L glucose; Capricorn Scientific GmbH, Ebsdorfergrund, Germany) with 40% FBS (Lot 2078409, Gibco, ThermoFisher Scientific, Darmstadt, Germany), 4 mM glutamine (200 Mm; Capricorn Scientific GmbH, Ebsdorfergrund, Germany) and 10% dimethyl sulfoxide (DMSO, Sigma Aldrich GmbH, Munich, Germany), and stored in liquid nitrogen.

Adipose-derived MSC from 5 horses euthanized for unrelated reasons were isolated from adipose tissue by digestion with collagenase and cryopreserved in liquid nitrogen as described previously (34). Their surface marker characterization is presented in the Supplementary File 1.

The equine PL used in this study was produced during a previous study and was prepared from whole blood from 19 horses (approved by regional council Giessen, A14/2019), using the buffy coat method (1). This pooled PL contained approximately 56 ng/ml IFN-γ, 49 ng/ml IL-10, 14 ng/ml IL-1β and 9 ng/ml TNF-α.

### Cell culture experiments

One million passage 1 MSC were thawed and pre-cultured in DMEM containing 10% FBS, 4 mM glutamine and 1% penicillin-streptomycin (10.000 U/ml Penicillin, 10 mg/ml Streptomycin; PAN-Biotech GmbH, Aidenach, Germany) under standard conditions (37 °C, humidified atmosphere, 5% CO_2_) in a 75 cm^2^ flask. Two days later, the MSC were detached and seeded in inserts (10,000 MSC per insert; TC-Inserts for 24 well plate, 0.4 μm pore, transparent, SARSTEDT AG & Co KG, Nümbrecht, Germany) in ultra-low-attachment (ULA) plates (Corning Costar 24 well clear flat bottom, NY, USA) with either 10% PL medium, 10% FBS medium or 10% FBS medium supplemented with 5 ng/ml recombinant equine IL-10 (Biomol GmbH, Hamburg, Germany) or 5 ng/ml IFN-γ (Biomol GmbH, Hamburg, Germany) for adaptation to the experimental conditions. These cytokine concentrations were chosen to reflect their anticipated initial concentrations in 10% PL medium. The basal medium remained the same DMEM with glutamine and penicillin-streptomycin.

Cryopreserved PBMC were thawed on the same day the MSC were seeded in the inserts, and precultured in DMEM containing 10% FBS, 4 mM glutamine and 1% penicillin-streptomycin, with stimulation by 50 ng/ml phorbol 12-myristate 13-acetate (PMA; Sigma Aldrich GmbH, Munich, Germany) and 1 μg/ml ionomycin (Sigma Aldrich GmbH, Munich, Germany) (35).

**Figure 1:**
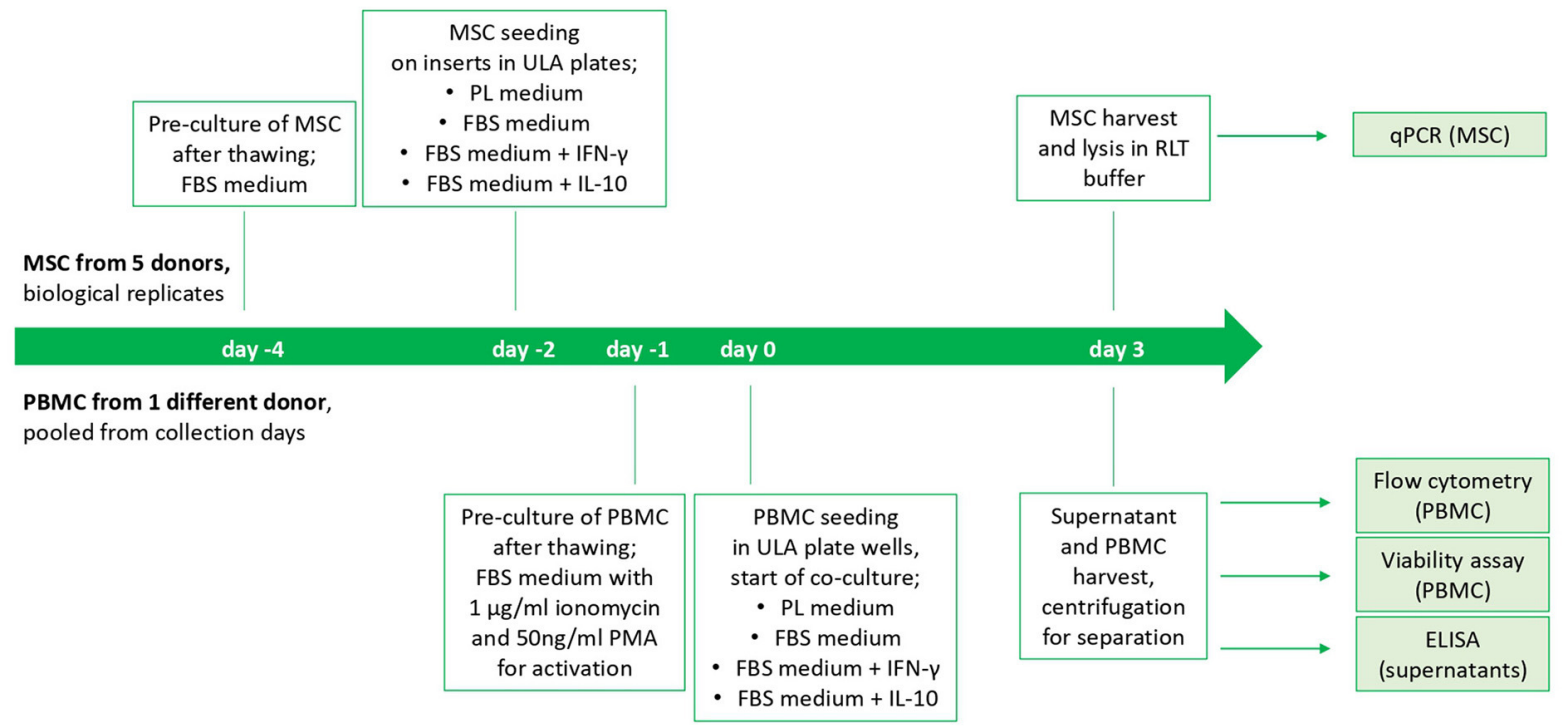
Study design and timeline of the cell culture experiment. The start is from beginning for each MSC donor. PBMC from different collection days of one horse were thawed and pooled.

On the following day, indirect co-cultures were prepared in transwells with the MSC-seeded inserts and the stimulated PBMC being added to the respective wells of the ULA plates (200,000 PBMC per well and 10,000 seeded MSC). Corresponding numbers of inserts and wells were left with PBMC and MSC cultured alone as control populations. Incubation of co-cultures and control cultures in the respective media as well as empty control media (PL, FBS, FBS+IFN- γ and FBS+IL-10, as described above) was performed for 3 days. The study design is displayed in Figure 1.

### ELISA

Cytokine concentrations were analyzed in all cell culture supernatants and in empty control media after the incubation of co-cultures for 3 days, using Equine DuoSet ELISA kits (R&D Systems, Minneapolis, MN, USA) for IFN-γ, TNF-α, IL-1β and IL-10 according to the manufacturer's instructions. The plates were read in an Infinite F50 plate reader and raw data were processed with the corresponding Magellan software (Tecan Ltd., Maennedorf, Switzerland). Samples with no measured cytokine content were assigned the value 0.

### qRT-PCR

MSC were analyzed for their relative gene expression of different cytokine-inducible factors related to immunoregulation and cell migration (Table 1) by quantitative real time PCR (qRT-PCR). Glyceraldehyde-3-phosphate dehydrogenase (GAPDH) and hypoxanthine phosphoribosyl transferase 1 (HPRT1) were used as reference genes. Using the RNeasy Mini Kit (Qiagen, Hilden, Germany), total RNA from each sample was isolated according to the manufacturer's instructions, including on-column DNase treatment, and converted to cDNA using the Reverse Transciptase RevertAid H Minus First Strand cDNA Synthesis Kit (Thermo Fisher). iQ SYBR Green Supermix (Bio-Rad Laboratories, Feldkirchen, Germany) and a qTowerG (Analytik Jena GmbH, Jena, Germany) were used to perform qRT-PCR. One μl (corresponding to 12.5 ng RNA) was used to analyze each target gene. Primers (Table 1) were defined using PrimerBlast (NCBI) and synthesized at IDT (Integrated DNA Technologies, Leuven, Belgium). Melting curve analysis was used to check for unspecific products. A dilution series of pooled experimental cDNA was used to determine the efficiencies of the PCR reactions. Relative gene expressions were calculated using the Pfaffl formula (36).

**Table 1:** Primers used for qRT-PCR.

**Gene**	**Primer sequence**	**Accession no**.	**PCR product (bp)**
eGAPDH	For: CGATGGTGAAGGTCGGAGTAAA Rev: TGGCGACAATATCCACTTTGC	NM_001163856.1	93
eHPRT1	For: ATTCTTTGCTGACCTGCTGGA Rev: AGGTCATCTCCACCAATCACTT	XM_023634464.1	147
eCOX2	For: GATCCTAAGCGAGGTCCAGC Rev: AGGCGCAGTTTATGCTGTCT	NM_001081775.2	101
eNOS2	For: GCGACCCTGAGCTCTTTGAA Rev: AGCTCCTGGAACCACTCGTA	NM_001081769.1	85
eIDO1	For: CTTCTTGTCTACGCAACGCC Rev: ACGCCTTCATAGAGCAGACC	XM_014736538.2	145
eTNFAIP6	For: TGCCTACTGCTACAACCCAC Rev: GTTTCCCTCCACTAGCAGACC	NM_001081906.1	127
eVCAM1	For: CTTACCTGTGCATCGTGACCT Rev: GTTTCCCTCCACTAGCAGACC	NM_001101650.1	119
eCXCR4	For: AGCAAAGTGACTCCGAGGAC Rev: CCACGTCATCCTCCGTGTAG	XM_005601469.3	117
MHC-II	For: CTTCGACAGCGACGTGGG Rev: AGTTGTGTCTGCAGTACGTGTC	NM_001142811.1	131
HIF1α	For: ACCATGCTTTGGACTCGGAT Rev: TGGCAAGCATCCTGTACTGT	XM_023627857.1	93

### Cell viability assay

The metabolic activity of PBMC was measured after co-cultivation on day 3 using a tetrazolium assay (MTS) according to the manufacturer's instructions (CellTiter 96^®^ Aqueous One Solution Cell Proliferation Assay, Promega, Mannheim, Germany). The mean absorbance, corrected by blank medium, was compared between PBMC cultured alone and PBMC in co-culture.

### Flow cytometry

Immunophenotypic analyses were performed in PBMC to assess the presence of Ki67 antigen as a proliferation marker and FoxP3 antigen as a marker for regulatory T cells.

First, PBMC were harvested from the ULA plates after 3 days of co-cultivation with MSC or without co-cultivation, respectively. After centrifugation at 500 g for 5 min at RT, the cell pellets were washed with 100 μl PBS each. The cells were then incubated with a fixable viability stain (1:1,000 in PBS; BD Horizon Fixable Viability Stain 620; BD Biosciences, Heidelberg, Germany) for 15 min protected from light. The cells were washed twice with PBS and incubated for 20 min at 4 °C in Fixation/Permeabilization Solution (BD Cytofix/Cytoperm Fixation/Permeabilization Kit). After washing again twice in 1x Perm/Wash Buffer (BD Cytofix/Cytoperm Fixation/Permeabilization Kit), resuspension of the cell pellet in 50 μl 10% goat serum in 1x Perm/Wash Buffer followed. After 15 min incubation at RT, 50 μl of Ki67 antibody dilution (1:1,000 in 1x Perm/Wash Buffer, final concentration 1:2,000; abcam ab281847, clone SP6, Alexa488; corresponding isotype control: Thermo Fisher #53-4616-82) were added and samples were incubated for 30 min at RT. After another 2 washing steps using 1x Perm/Wash Buffer, cells were resuspended in 50 μl 1x Perm/Wash Buffer containing 5% rat serum and then incubated again for 15 min at 4 °C. Following that, 50 μl of FoxP3 antibody dilution (1:10 in 1x Perm/Wash Buffer, final concentration 1:20; Thermo Fisher, clone FJK-16s, PE; corresponding isotype control: BioLegend, Rat IgG2a) were added and samples were incubated at 4 °C for 30 min. Cells were then washed again twice with 1x Perm/Wash Buffer and fixed in 2% paraformaldehyde in PBS for 15 min at RT. Fixed cells were washed twice with PBS and stored overnight in PBS containing 3% FBS at 4°C.

Flow cytometric measurements of 100,000 events per sample were performed on a BD Accuri^TM^ C6 Plus Personal Flow Cytometer. Data was analyzed using FlowJo^TM^ v 10.7 software (FlowJo, LLC, BD Biosciences, Ashland, OR, USA). Lymphocytes were gated in an FSC-A vs. SSC-A dot plot. Dead cells were excluded based on staining with the BD Horizon Fixable Viability Stain 620. Marker expression gates were set based on the corresponding isotype controls.

### Statistical analysis

SPSS statistics version 29.0.1.1 (IBM, Ehningen, Germany) was used for comparisons between experimental groups. Non-parametric group tests for paired samples and, in case of significance, the respective *post-hoc* tests were computed, with the *post-hoc* tests being subject to Bonferroni corrections. Differences were considered significant at *p* < 0.05. Graphpad Prism 8 (GraphPad Software, Boston, USA) was used for graphical presentation of data.

## Results

### Cytokine concentrations after cell culture

**Figure 2:**
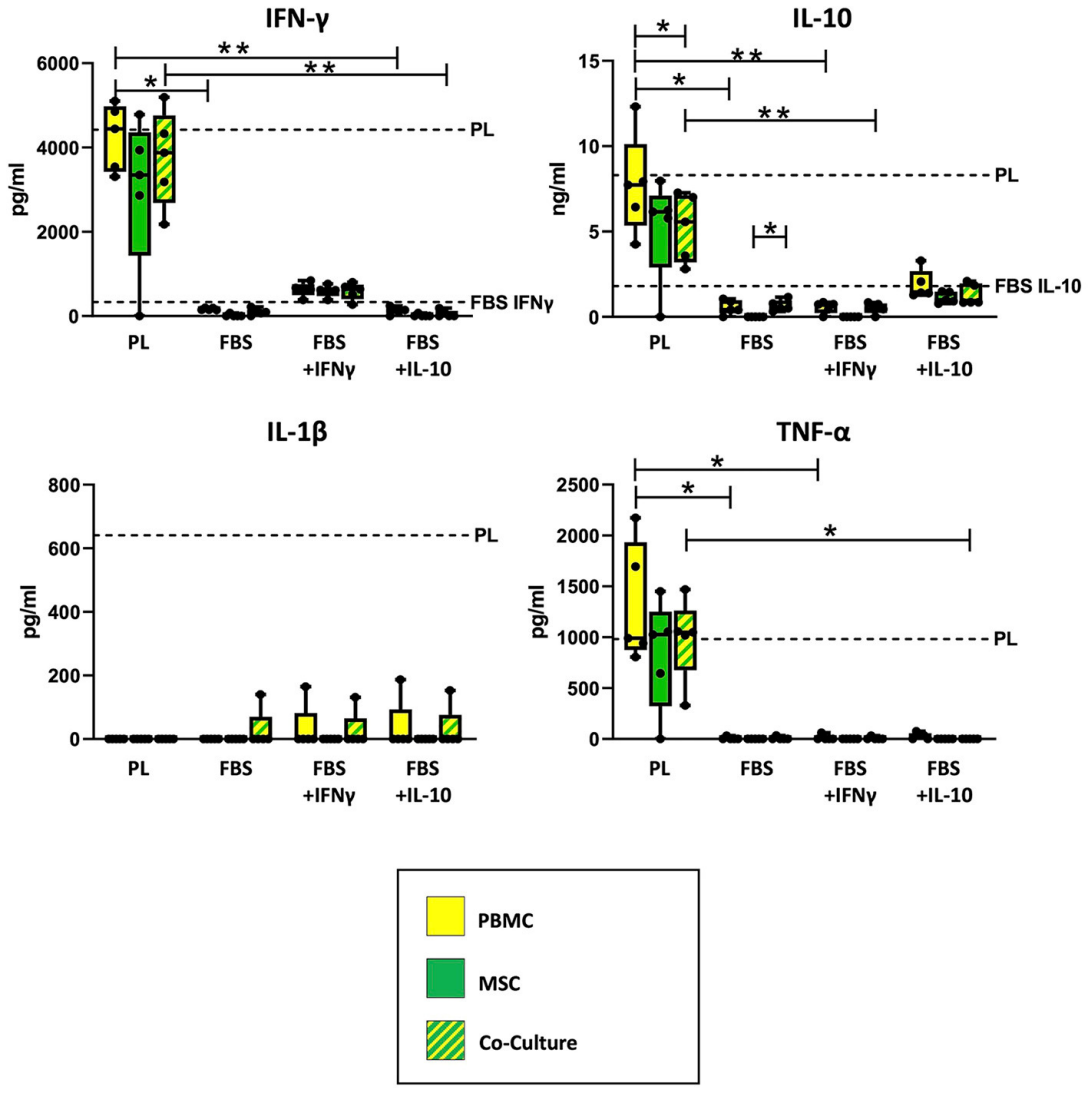
Cytokine concentrations measured in supernatants and empty control culture media. IFN-γ, IL-10, IL-1β and TNF-α concentrations were determined in supernatants from MSC, PBMC and indirect co-cultures as well as in empty control media using equine ELISA kits after 3 days of incubation. The culture medium was either supplemented with 10% platelet lysate (PL), 10% fetal bovine serum (FBS), 10% FBS and 5 ng/ml equine IFN-γ (FBS + IFNγ) or 10% FBS and 5 ng/ml equine IL-10 (FBS + IL-10). The whiskers extend to minimum and maximum values and the points display the data from the individual experiments. The dashed lines show the concentration of the respective cytokine in empty control medium after the incubation time. For media which are not indicated by a dashed line, the respective equine cytokine was not detected. Asterisks indicate significant differences between groups, either between the same cells in different media, or between different cells in the same medium (*p < 0.05; **p < 0.01). Data were obtained in independent experiments with n = 5 different MSC donors.

As shown in Figure 2, ELISA measurements revealed that empty PL control medium still contained approximately the same concentrations of IFN-γ and IL-10, even after the co-culture incubation time of 3 days. The concentrations of IL-1β and TNF-α in PL control medium had decreased over the incubation period but were still measurable. In contrast, none of the cytokines were detected in empty FBS control medium without equine cytokine supplementation, and the supplemented cytokines IFN-γ and IL-10 had strongly decreased in their concentration over the incubation period.

Correspondingly, the measured cytokine concentrations in PBMC and co-culture supernatants differed significantly between PL and FBS media, except for IL-1β (IFN-γ: *p* = 0.009 for PBMC in PL vs. FBS, *p* = 0.02 for PBMC in PL vs. FBS+IL-10, *p* = 0.002 for co-cultures in PL vs. FBS+IL-10; IL-10: *p* = 0.02 for PBMC in PL vs. FBS, *p* = 0.009 for PBMC in PL vs. FBS+IFN-γ, *p* = 0.01 for co-cultures in PL vs. FBS+IFN-γ; TNF-α: *p* < 0.05 for PBMC in PL vs. FBS or vs. FBS+IFN-γ, *p* = 0.042 for co-cultures in PL vs. FBS+IL-10). The analogous differences were not significant for MSC supernatants, which overall contained lower concentrations of cytokines compared to PBMC and co-cultures.

Regarding IFN-γ, the supernatants from PBMC, MSC and co-cultures incubated in PL medium had similar (PBMC) or slightly lower (MSC) concentrations as their control culture medium, indicating that IFN-γ release from the cells and IFN-γ binding and degradation during the incubation were quite well balanced. The supernatants from cells cultivated in FBS+INF-γ medium showed lower IFN-γ concentrations as compared to PL medium but higher IFN-γ concentrations than their own control medium, suggesting that the cells had produced and released new IFN-γ. In contrast, in supernatants from cells cultured in FBS media without IFN-γ supplementation, no or very low IFN-γ concentrations were detected. When MSC were cultured alone, most samples did not release any detectable IFN-γ. There were, however, no significant differences between cell types within each medium.

For IL-10, again there were high concentrations of IL-10 in PL medium cell culture supernatants, for PBMC near its concentration in PL control medium but for MSC and co-cultures lower than in PL control medium (*p* = 0.043), indicating that the latter bound and degraded more IL-10 than they released. Lower concentrations of IL-10 were found in FBS+IL-10 medium supernatants, again for PBMC close to the control medium and for MSC and co-cultures below that. Very low concentrations of IL-10 were detected in supernatants from PBMC or co-cultures in FBS media without IL-10 supplementation. Again, MSC cultured alone did not release IL-10. Based on that, in FBS medium, IL-10 release was significantly higher in co-cultures than in MSC alone (*p* = 0.042).

TNF-α was present in all supernatants from cells cultured with PL medium, approximately at the same concentration as in the PL control medium. In FBS media, TNF-α was never found in MSC supernatants and only in few supernatant samples from PBMC and co-cultures. In FBS+IL-10 medium, none of the co-cultures released TNF-α.

IL-1β was not detected in most supernatants, despite being found in the PL control medium, which indicates it was completely bound or degraded by PBMC and MSC. However, there were some outliers with detectable IL-1β levels in the co-culture with FBS medium, and in the co-cultures but also PBMC cultures with FBS+IFN-γ and FBS+IL-10 medium.

### Immunoregulatory gene expression in MSC

**Figure 3:**
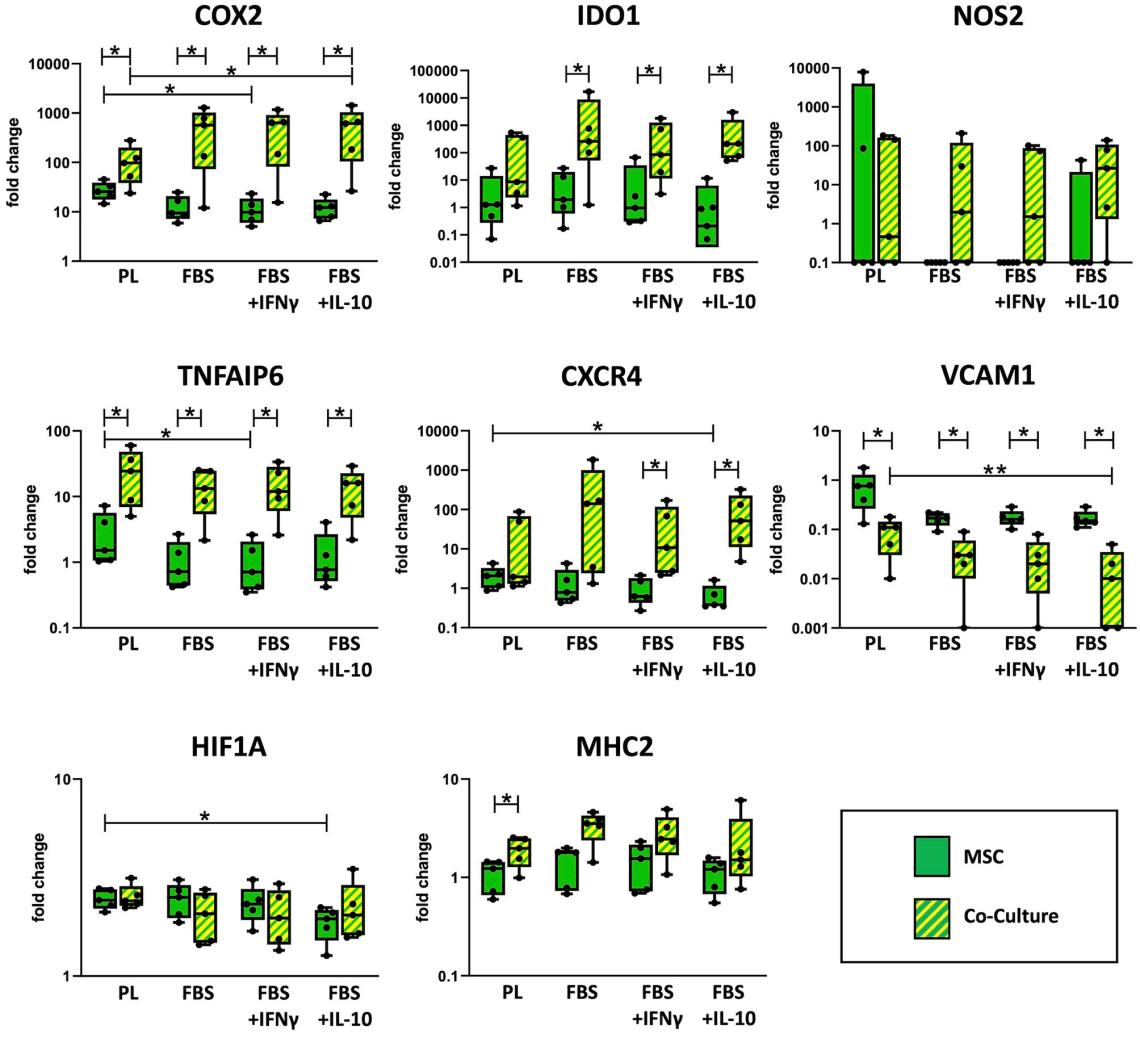
Relative gene expression in MSC cultured alone or in co-culture with PBMC. Gene expression in MSC was analyzed by qRT-PCR after 3 days of incubation in the respective culture medium, with or without PBMC. The culture medium was either supplemented with 10% platelet lysate (PL), 10% fetal bovine serum (FBS), 10% FBS and 5 ng/ml IFN-γ (FBS+IFNγ) or 10% FBS and 5 ng/ml IL-10 (FBS+IL-10). The whiskers extend to minimum and maximum values and the points display the data from the individual experiments. Asterisks indicate significant differences between groups, either between the same cells in different media, or between different cells in the same medium (*p < 0.05; **p < 0.01). Data were obtained in independent experiments with n = 5 different MSC donors.

As shown in Figure 3, co-culture upregulated the MSC gene expression of the cyclooxygenase COX2, TNFAIP6 (encoding for the TNF-α-stimulated TSG6), the indoleamine 2,3-dioxygenase IDO1, the chemokine receptor CXCR4 and the major histocompatibility complex MHC2 compared with MSC cultured alone. For COX2 and TNFAIP6, this difference was significant throughout all media (*p* = 0.043). For IDO1, significance was only reached in FBS media (*p* ≤ 0.043) but not in PL medium, and for CXCR4, differences were only significant in FBS media supplemented with the cytokines IFN-γ or IL-10 (*p* = 0.043) but not in PL or standard FBS media. In contrast, for MHC2, the upregulation in co-culture was significant in PL medium (*p* = 0.043) but not in the FBS media.

On the other hand, co-culture downregulated MSC gene expression of the vascular cell adhesion molecule VCAM1. This was again significant in all media (*p* < 0.043), with the overall highest VCAM1 expression being observed in MSC cultured alone in PL medium.

Differences in MSC immunoregulatory gene expression were also found across the different media. Overall, the expression of several immunoregulatory genes was higher in MSC cultured in PL medium as compared to FBS media, although this effect was reversed for some genes by the presence of PBMC. TNFAIP6 and VCAM1 were expressed at higher levels in PL medium, irrespective of the MSC being cultured alone or with PBMC (*p* = 0.009 for TNFAIP6, PL vs. FBS+IFN-γ in MSC alone and for VCAM1, PL vs. FBS+IL-10 in co-cultured MSC). For COX2 and CXCR4 expression, the effects of culture media supplements were different between MSC cultured alone and MSC co-cultured with PBMC. In MSC alone, both COX2 and CXCR4 were expressed at higher levels when MSC were cultured in PL medium compared to FBS media (*p* = 0.02 for COX2, PL vs. FBS+IFN-γ; *p* = 0.004 for CXCR4, PL vs. FBS+IL-10). Contrarily, in MSC that were co-cultured with PBMC, both COX2 and CXCR4 were expressed at lower levels in PL medium as compared to FBS media (*p* = 0.042 for COX2, PL vs. FBS+IL-10).

The expression of hypoxia-inducible factor HIF1A was not altered much between culture conditions. Nevertheless, its expression was lowest in MSC cultured alone in FBS+IL-10 medium (*p* = 0.042 compared to MSC alone in PL medium). The inducible nitric oxide synthase NOS2 was expressed at very low levels in all samples except for one outlier within the group of MSC cultured alone in PL medium. No significant differences were observed between any of the groups regarding NOS2 expression.

### Regulatory effects on PBMC

**Figure 4:**
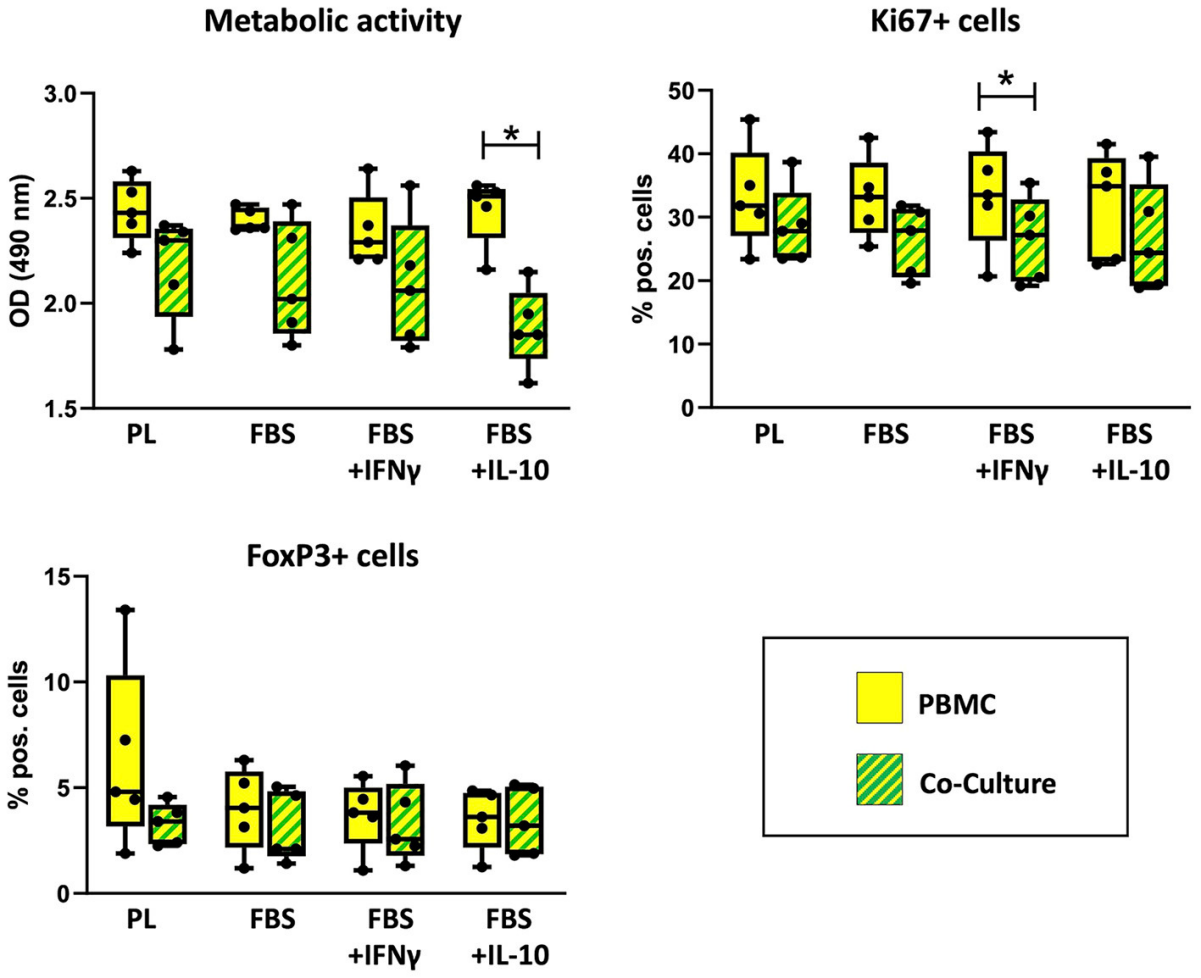
Viability and percentages of FoxP+ cells in PBMC cultured alone or with MSC. Metabolic activity, Ki67+ proliferative cells and FoxP+ regulatory cells were analyzed by MTS assay and flow cytometry after 3 days of incubation in the respective culture medium, with or without MSC. The culture medium was either supplemented with 10% platelet lysate (PL), 10% fetal bovine serum (FBS), 10% FBS and 5 ng/ml IFN-γ (FBS+IFNγ) or 10% FBS and 5 ng/ml IL-10 (FBS+IL-10). The whiskers extend to minimum and maximum values and the points display the data from the individual experiments. Asterisks indicate significant differences between PBMC alone and co-cultures in the same medium (*p < 0.05). Data were obtained in independent experiments with n = 5 different MSC donors.

PBMC viability and flow cytometry results are shown in Figure 4. The metabolic activity of PBMC, determined by MTS assay, was decreased after co-culture in comparison to the corresponding PBMC cultured alone. This effect was significant in FBS medium supplemented with IL-10 (*p* = 0.043). The percentage of Ki67+ PBMC, reflecting proliferative cells as measured by flow cytometry, had decreased after co-culture with MSC. This difference was significant in FBS medium supplemented with IFN-γ (*p* = 0.043). For both parameters, no significant difference was found between corresponding cell cultures in different media.

The percentages of regulatory T cells expressing the marker FoxP3 were similar in co-cultures and PBMC alone. Maximum percentages of FoxP3+ PBMC were found in PBMC cultured alone in PL medium, but this was not consistent between experiments. Significant differences were neither found between PBMC alone and PBMC in co-culture, nor between different media.

## Discussion

The main goal of this study was to determine whether PL medium can improve MSC immunomodulatory characteristics, with the perspective that it could replace conventional FBS medium supplemented with cytokines for MSC priming. In this line, we also tested if naturally occurring cytokine concentrations, either in the PL or supplemented, would act synergistically with the MSC to modulate PBMC.

Priming with inflammatory cytokines during cell culture plays a decisive role in the immunomodulatory potency of MSC. For example, Cuerquis et al. (18) and Hemeda et al. (37) described that T cell proliferation was better inhibited by MSC primed with IFN-γ and TNF-α. Furthermore, various studies have shown that the concentration of supplemented cytokines has a strong influence on the immunomodulatory effect of MSC. In some studies, a highly inflammatory environment, represented by incubation with TNF-α, IFN-γ or IL-2 (20 ng/ml), promoted the immunosuppressive effect of MSC (38), while in a low-inflammatory environment, represented by 2 ng/ml of TNF-α or IFN-γ, MSC can enhance inflammation due to insufficient production of iNOS (39). However, this effect apparently depends on the nature of the artificial inflammation and type of cell culture model, as we had previously observed opposite effects in direct co-cultures of non-primed equine MSC and differentially activated leukocytes (35).

Equine PL contains a naturally composed mix of cytokines, and therefore might mimic inflammation to an extent that beneficially impacts on MSC. However, donor effects on PL composition should be considered: in a previous study, we found that cytokine levels in PL were subject to high individual variation (4). Particularly horses with increased alkaline phosphatase, creatinine and lactate dehydrogenase values also had an increased concentration of IFN-γ, TNF-α, IL-4, IL-6, IL-10 and IL-1β in their blood products including PL. For cell culture experiments in the current study, we used pooled PL from the horses included in the previous study to level out the high individual variability.

To assess the stability of the cytokines, present in PL or supplemented to FBS media, as well as to shed light on possible cytokine release by MSC, PBMC and their co-cultures, ELISA measurements of media and supernatants were performed after the incubation period. Regarding the detection of cytokines in standard FBS medium, it needs to be acknowledged that the equine ELISA kits chosen for the current study are not suitable to accurately detect the respective bovine cytokines. Therefore, the conclusion that these cytokines were completely absent in empty FBS medium cannot be drawn. Regarding equine cytokines, the supplemented IFN-γ and IL-10 had strongly decreased during incubation, while they were surprisingly stable in PL control medium. This suggests that PL, in contrast to FBS, also contains molecules to protect its cytokines, either by higher availability of carrier proteins or by inhibiting degradation enzymes. However, this does not account for all cytokines to the same extent. Other than IFN-γ and IL-10, TNF-α and IL-1β in PL control medium had decreased during incubation. Interestingly, IL-1β was not detectable at all after cells had been incubated in the PL medium.

ELISA further revealed that PBMC released IFN-γ, IL-10, IL-1β and TNF-α. Here, it should be acknowledged that for IL-1β and TNF-α, this was inconsistent between experiments and failed to markedly elevate the supernatant concentrations. MSC alone regularly did not release cytokines at detectable levels. Only with IFN-γ supplementation to FBS medium, some additional release of IFN-γ by MSC alone was evident. However, it was interesting that in the presence of MSC, cytokine concentrations in PL had decreased as compared to the empty control medium, most notably regarding IL-10. On the one hand, this might be due to increased cytokine degradation by MSC-derived enzymes such as matrix metalloproteinases (40, 41). On the other hand, we believe that the supernatant cytokine concentrations also decreased due to cytokine binding to their respective receptors on the MSC. Both options would also explain the loss of detectable IL-1β in supernatants. Binding to the appropriate receptor cannot be finally proven by the current experiments but appears plausible based on the current evidence for downstream gene regulation.

Investigating the expression of immunoregulatory genes in MSC in response to the different media and to co-culture with PBMC yielded interesting results. Given that all these genes had previously been shown to be upregulated in the presence of cytokines (15, 42–45), we had expected to see an upregulation of several of them in PL medium as well as in FBS medium supplemented with either IFN-γ or IL-10. Interestingly, however, this was not the case in cytokine-supplemented FBS media, while in PL medium, several genes including COX2, TNFAIP6, VCAM1, CXCR4 and HIF1A, were expressed at higher levels. At the same time, favorably, MHC2 expression was not increased in PL medium when MSC were cultured alone. The lacking response in gene expression to artificial cytokine supplementation in comparison to the naturally present cytokines in PL could be due to the following reasons: First, the concerted action of at least two different cytokines, among them often IFN-γ, is required to induce changes in the target genes (15, 45), which is the case in PL medium. Second, as revealed by the ELISA measurements, IFN-γ and IL-10 were more stable in PL medium, which has likely led to prolonged potency. Both arguments underline that PL is highly suitable to deliver a potent mix of cytokines.

Besides the upregulation of several immunomodulatory genes in the presence of PL, the activation of immunoregulatory mechanisms in equine MSC in the presence of stimulated PBMC was demonstrated by qPCR analysis. This is in line with previous studies demonstrating increased production of anti-inflammatory mediators such as IL-10 by MSC when co-cultured with peripheral blood leukocytes (35), as well as increased IDO expression and PGE2 release upon co-culture with lymphocytes (46). In the current study, the expression of COX2, TNFAIP6, IDO1, CXCR4 and MHC2 was higher in co-culture, with most impressive changes in COX2 expression. In contrast, VCAM1 was downregulated as compared to MSC cultured alone. Downregulation of VCAM1 in PL medium, however, did not reach levels as low as those observed in FBS media.

The target genes chosen in this study are not only induced by cytokines but are also indicative of MSC potency. The COX2/PGE2 pathway has been repeatedly related to immunosuppressive potency, e.g. with respect to regulating lymphocyte suppression, dendritic cell maturation and macrophage phenotype switch (24, 47). In this line, the strong COX2 upregulation in co-cultures is likely part of the mechanism inducing the here observed suppression of PBMC. TSG6, encoded by TNFAIP6, is significantly involved in the macrophage shift (17, 25). Moreover, TSG6 has been suggested as overall biomarker for MSC potency due to the positive correlation between TSG6 levels and potency measures *in vitro* and *in vivo* (48). TNFAIP6 was among the genes that were more expressed in presence of PL, which could be the consequence of TNF-α being present in PL but presumably not in FBS media. Both CXCR4 and VCAM1 are related to the MSC capability of homing, for which CXCR4 appears to be unambiguously beneficial but VCAM1 has also been reported to inhibit migratory potential when highly expressed (44, 49). Therefore, upregulation of CXCR4 and downregulation of VCAM1 as observed in the current study might together improve MSC homing. VCAM1, however, in that context better known as CD106, has on the other hand been suggested as a marker for highly immunomodulatory MSC (50). With respect to the current results, the downregulation of VCAM1 in co-cultures is in accordance with the previous finding that this adhesion molecule would only be secreted with direct cell-cell contact between MSC and lymphocytes (51), which was prevented by the transwell inserts in our study. IDO1 serves as one of the important factors for T cell suppression and reduction of their proliferation (16) and is also relevant for M2 macrophage differentiation (52). The current observation of IDO1 upregulation in the presence of activated PBMC is in accordance with previous findings (46).

In contrast to the aforementioned genes and their proteins, increased MHC2 expression must be considered with caution as increased MHC2 expression in MSC is traditionally related to stronger immune responses to the MSC, particularly when haplotypes are mismatched (32, 53). In accordance with previous studies (46, 54), MHC2 expression increased in co-cultures in the current work. Nevertheless, it was not increased by the presence of PL alone, despite the high IFN-γ concentrations in PL medium. Upregulation of MHC2 in MSC depends on a variety of factors. For example, IFN-γ-induced upregulation was reduced by TGF-β (55), which is also part of PL composition (1). In any case, MHC2 upregulation alone does not necessarily disqualify the MSC. Likely depending on the overall result of inflammatory activation, MSC with upregulated MHC2 but lacking co-stimulatory CD86 were potent in the adaptive regulation of T cells *in vitro* (56).

In the current study, PBMC proliferation was always decreased in the presence of MSC, with the same trend in PL and FBS media, demonstrating their immunoregulatory potential on a functional level. Nevertheless, this reached significance only in the FBS media supplemented with cytokines, but surprisingly not in PL medium. MSC influence macrophages, monocytes and dendritic cells by keeping them in an immature anti-inflammatory state in inflammatory areas and thus preventing the activation of T cells, while promoting the differentiation of regulatory T cells (57). In previous studies, MSC promoted the differentiation and activation of CD4+/CD25+/FoxP3+ T cells and IL10+ Treg cells (58, 59). However, in the current study, these results could not be reproduced. There were neither significant differences between co-cultures and PBMC cultured alone nor between the different media regarding the percentage of FoxP3+ cells. One possible explanation for this is that several studies- albeit not all- showed that the induction of FoxP3+ Treg cells by MSC requires direct cell-cell contact (60, 61). Based on the result that, although inconsistent, the highest percentages of FoxP3+ cells were found in PBMC in PL medium without MSC in the current study, it may be worth to further investigate this issue.

In this study, we focused on the immunomodulatory capabilities of equine MSC in PL and FBS media. Various studies, including our own, already showed that the proliferative capacity, differentiation potential and immunophenotype of equine MSC are preserved in PL medium (1, 62, 63). The current data complement these previous findings by demonstrating that the cytokines in PL could provide long-term stimulation and promote MSC immunoregulatory gene expression without any negative effects being observed. However, on functional level, MSC in PL medium did not outperform MSC in FBS media in the current experimental setup. Therefore, the current study adds to the perception that PL is a viable and, in many respects, superior alternative to FBS, while PL-based MSC priming is yet to be further investigated and optimized.

## Data availability statement

The original contributions presented in the study are included in the article/Supplementary material, further inquiries can be directed to the corresponding author.

## Ethics statement

The animal studies were approved by the Regional Council Giessen, A17/2018. The studies were conducted in accordance with the local legislation and institutional requirements. Written informed consent was obtained from the owners for the participation of their animals in this study.

## Author contributions

JM: Conceptualization, Data curation, Formal analysis, Investigation, Methodology, Project administration, Software, Validation, Visualization, Writing—original draft, Writing—review & editing. SN: Conceptualization, Data curation, Methodology, Validation, Writing—review & editing. KF: Supervision, Validation, Writing—review & editing. AH: Methodology, Resources, Writing—review & editing. JB: Conceptualization, Methodology, Project administration, Supervision, Validation, Writing—review & editing, Resources.

## Supplementary material

Supplementary File 1 Immunophenotype of equine adipose-derived MSC.
