# Supplementary material for: Impact of platelet lysate on immunoregulatory characteristics of equine mesenchymal stromal cells
Source: Front Vet Sci. 2024 Apr 24;11:1385395. doi: 10.3389/fvets.2024.1385395 (PMC11079816; doi:10.3389/fvets.2024.1385395)

Supplement to Moellerberndt et al.:

Immunophenotype of equine adipose-derived MSC

MSC were harvested, stained with fluorescent-labeled monoclonal antibodies (see Table) and analyzed using a Cytoflex flow cytometer (Beckman Coulter). The figure displays representative histograms of surface marker stainings (red) and the corresponding isotype controls (blue).

Antibody	Clone	Reactivity	Company	Dilution
**CD29-Alexa488**	TS2/16	Anti-human	Biolegend, San Diego, California	1:20
**CD44-APC**	IM7	Anti-rat	BD, Franklin Lakes, New Jersey	1:100
**CD73-Alexa488**	606112	Anti-human	R&D, Minneapolis, Minnesota	1:40
**CD90-APC**	5E10	Anti-human	BD, Franklin Lakes, New Jersey	1:100
**CD105-PE**	SN6	Anti-human	Serotec, Kidlington, UK	1:10
**CD14-APC**	134620	Anti-human	R&D, Minneapolis, Minnesota	1:50
**CD34-FITC**	43A1	Anti-human	Santa Cruz, Santa Cruz, California	1:100
**CD45-Alexa488**	F10-89-4	Anti-human	Serotec, Kidlington, UK	1:5
**CD79α-PE**	HM57	Anti-human	Serotec, Kidlington, UK	1:2,5
**MHC II-PE**	CVS20	Anti-equine	Serotec, Kidlington, UK	1:50

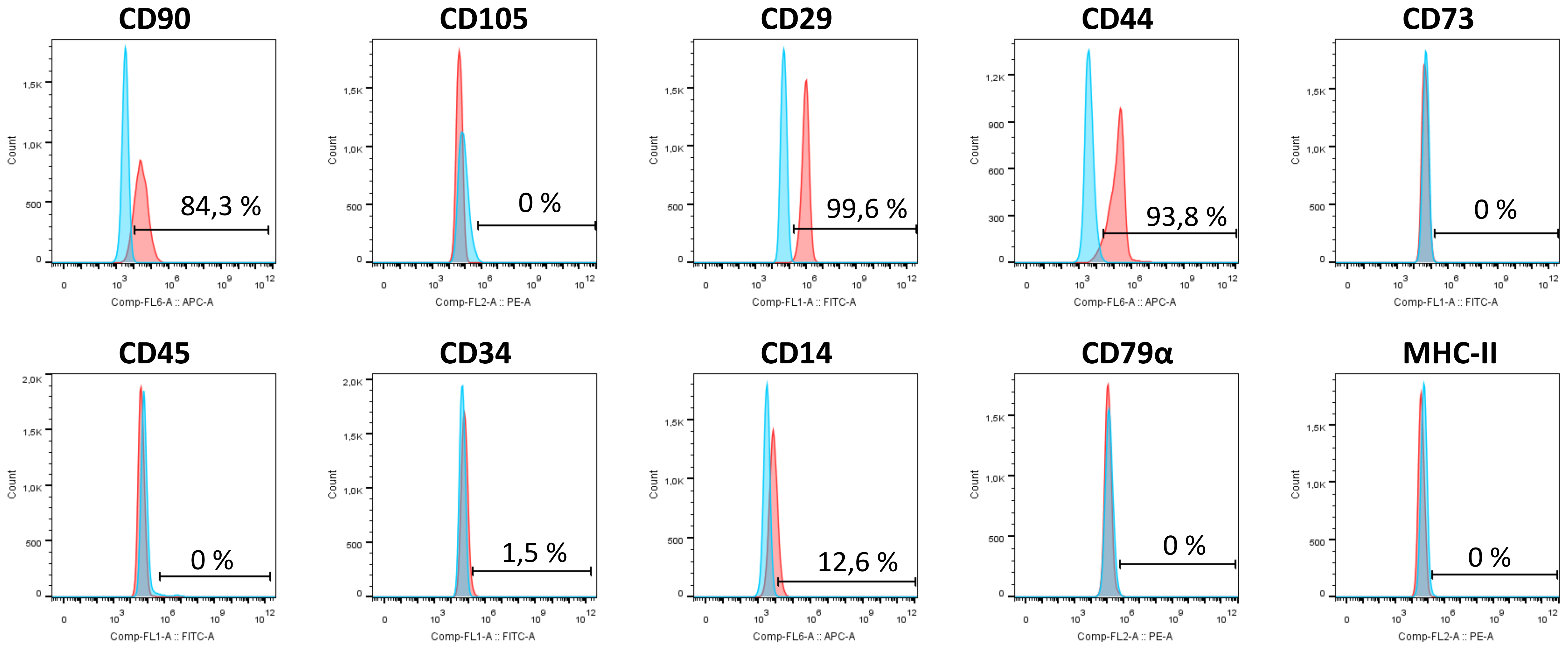

Supplement: Supplementary File 1 — Immunophenotype of equine adipose-derived MSC. [file Data_Sheet_1.PDF]
